# The Association Between Metabolic Syndrome and Prostate Cancer Risk: A Large-Scale Investigation and Study of Chinese

**DOI:** 10.3389/fendo.2022.787268

**Published:** 2022-05-20

**Authors:** Xiaoshuai Gao, Ruicen Li, Tao Jin, Huairong Tang

**Affiliations:** ^1^Department of Urology, Institute of Urology (Laboratory of Reconstructive Urology), West China Hospital, Sichuan University, Chengdu, China; ^2^Department of Health Management Center, West China Hospital, Sichuan University, Chengdu, China

**Keywords:** metabolic syndrome, prostate cancer, prostate-specific antigen, hypertension, obesity

## Abstract

**Background:**

To investigate the association between metabolic syndrome (MetS) and its components and prostate cancer (PCa).

**Methods:**

This study enrolled 482 943 consecutive men who underwent routine health checkups at the Health Management Center of West China Hospital Between 2010 and 2017. For patients with elevated prostate-specific antigen (PSA) levels or color Doppler ultrasound indicating abnormal prostates, we recommended prostate puncture and follow-up. We used the chi-square test and independent t-test for categorical variables and continuous variables, respectively. We used logistic regression analysis to evaluate the effects of MetS and its components on prostate cancer risk.

**Results:**

We found that the incidence of PCa in Chinese men over 40 years of age was 0.1%. Among the 85882 participants, 31.5% (27016/85882) of the patients were diagnosed with MetS. PCa was associated with older age, higher PSA levels, lighter weight and shorter height, hypertension, elevated fasting blood glucose (FBG) and HDL cholesterol level, lower triglycerides. After excluded the interference of other factors in multivariate logistic analysis, we found that MetS, hypertension, hyperlipidemia, hyperglycemia, and obesity were not related to the risk of PCa. High age and PSA levels were risk factors for prostate cancer.

**Conclusions:**

High age and PSA levels were risk factors for prostate cancer. MetS, hypertension, hyperlipidemia, hyperglycemia, and obesity were not related to the risk of PCa.

## Introduction

Prostate cancer (PCa) is the second most common tumor in males, and the fifth leading cause of cancer-related mortality (1). An international cancer study revealed that PCa incidence rates are increasing in 32 of 40 countries, while rates are relatively stable in the remaining eight countries (2). An increasing incidence of PCa is inseparable from the Western high-fat diet, sedentary lifestyle and obesity (2).

Metabolic syndrome (MetS) is a metabolic disorder that includes at least three of the following five items: hypertension, hyperglycemia, obesity, an elevated triglyceride level and decreased high-density lipoprotein cholesterol (HDL-C) (3). Over the past few decades, MetS is increasing worldwide because of sedentary lifestyles and excessive carbohydrate intake (4–6). Although the major complication of MetS is cardiovascular disease, a few studies have shown that it increases the risk for PCa (4).

The relationship between MetS and PCa is controversial. Some studies have shown that MetS increases the risk for PCa (4, 7), while some studies have indicated that MetS reduces the risk for PCa (8, 9), and some studies have shown no relationship between MetS and PCa (10). A recent meta-analysis showed that although MetS had little effect on the incidence of PCa, it significantly increased the incidence of high-grade PCa (11). Another high-quality study showed that the relationship between MetS and PCa is affected by race and country. In European countries, MetS increases the risk for PCa, but it has no significant effect on PCa risk in Asian countries and the United States (10).

To better explore the relationship between MetS and PCa, we conducted a large-scale study on patients undergoing health checkups in China. For patients with elevated prostate-specific antigen (PSA) levels and magnetic resonance imaging (MRI) indicating abnormal prostates, we recommended prostate puncture and follow-up.

## Materials and Methods

### Patients and Include Data

This study included 482 943 consecutive men who underwent routine health checkups at the Health Management Center of West China Hospital between 2010 and 2017. For people who participated in multiple health checkups, we only included data from the first visit. The screening process for this study was as follows: 315 836 patients were excluded for duplicate data, 687 were excluded for history of PCa or prostatectomy, 11278 were excluded for missing important data and 69260 were excluded as they were younger than 40 years of age. Finally, 85882 patients were included in the analysis, and 102 patients were diagnosed with PCa.

The included data were as follows: age, weight, height, body mass index (BMI), waist circumference, diastolic blood pressure (DBP), systolic blood pressure (SBP), HDL-C, triglycerides, fasting blood glucose (FBG), medical history and PSA. For patients with elevated PSA levels or color Doppler ultrasound indicating abnormal prostates, we recommended prostate MRI examination. If MRI suggests that there is a prostate mass, it is recommended to perform a prostate puncture and tracked the Gleason score at our hospital.

### Definition of the MetS

MetS was defined according to the multidisciplinary international joint declaration (3). Patients were diagnosed with MetS when they had at least three of the following presentations: (1) obesity, waist circumference ≥ 85 cm; (2) hypertension, elevated blood pressure (systolic ≥ 130 mmHg and/or diastolic ≥ 85 mmHg; (3) dyslipidemia, HDL-C < 40 mg/dL (1.0 mmol/L); (4) dyslipidemia, triglyceride levels ≥ 150 mg/dL (1.7 mmol/L); and (5) hyperglycemia, FBG ≥ 100 mg/dL (5.6 mmol/L).

### Statistical Analyses

All statistical analyses were performed with IBM SPSS software version 23.0 for Mac. Continuous variables are presented as the median (range) and were compared by an independent t-test. Categorical variables were expressed as rates and compared by the chi-squared test. Univariate logistic regression analysis was used to calculate odds ratios (ORs) and 95% confidence intervals (CIs). Multivariate logistic regression analysis was used for P < 0.05. All P values were two-sided, and P < 0.05 indicated that the difference was statistically significant.

## Results

### Baseline Patient Characteristics

The demographic characteristics of the participants are presented in **Table 1**. Overall, 0.1% (102/85882) of participants were newly diagnosed with PCa. Among the 85882 participants, 31.5% (27016/85882) of the patients were diagnosed with MetS. PCa was associated with older age, higher PSA levels, lighter weight and shorter height, hypertension, elevated fasting blood glucose (FBG) and HDL cholesterol level, lower triglycerides.

**Table 1:** Baseline characteristics of patients according to the diagnosis of PCa.

Characteristics	Overall	Without PCa	With PCa	P
Men (number)	85882	85780 (99.9)	102 (0.1)	
Age (years)	49 (40–100)	49 (40-100)	70 (44-85)	<0.001
Weight (kg)	69 (32-130)	69 (32-130)	67 (43-87)	0.012
Height (cm)	167 (132-202)	167 (132-202)	165 (153-180)	0.001
BMI (kg/m^2^)	24.77 (13.36-41.52)	24.77 (13.36-41.52)	24.425 (14.88-32.85)	0.389
Obesity, n (%)				
Waist circumference ≥85cm	49314 (57.4)	49256 (57.4)	58 (56.9)	0.909
Waist circumference<85cm	36568 (42.6)	36524 (42.6)	44 (43.1)	
High blood pressure, n (%)				
SBP ≥130 or DBP ≥85	33697 (39.2)	33640 (39.2)	57 (55.9)	0.001
SBP <130 and DBP <85	52185 (60.8)	52140 (60.8)	45 (44.1)	
High triglycerides, n (%)				
≥1.7mmol/L	36975 (43.1)	36943 (43.1)	32 (31.4)	0.017
<1.7mmol/L	48907 (56.9)	48837 (56.9)	70 (68.6)	
High FBG level, n (%)				
≥5.6mmol/L	26433 (30.8)	26391 (30.8)	42 (41.2)	0.023
<5.6mmol/L	59449 (69.2)	59389 (69.2)	60 (58.5)	
Low HDL cholesterol level, n (%)				
<1.0mmol/L	12673 (14.8)	12666 (14.8)	7 (6.9)	0.025
≥1.0mmol/L	73209 (85.2)	73114 (85.2)	95 (93.1)	
MetS (%)				
With MetS	27016 (31.5)	26991 (31.5)	25 (24.5)	0.131
Without MetS	58866 (68.5)	58789 (68.5)	77 (75.5)	
Serum PSA (ng/mL)	0.80 (0.00-148.39)	0.80 (0.00-148.39)	8.31 (0.45-99.90)	<0.001

MetS, metabolic syndrome; PSA, prostate specific antigen; BMI, Body mass index; SBP, systolic blood pressure; DBP, diastolic blood pressure; FBG, fasting blood glucose; HDL, high-density lipoprotein. The chi-square test and independent t-test were respectively used for categorical variables and continuous variables. Obesity is defined as waist circumference ≥85 cm. P < 0.05 was considered significant.

The definition of MetS is adapted from the modified National Cholesterol Education Programme–Adult Treatment Panel (NCEP-ATP) criteria.

### Risk Factors for Prostate Cancer

As shown in **Table 2**, in the univariate analysis, older age, higher hypertriglyceridemia, lower HDL-C and higher PSA levels increased the risk for PCa. However, hyperglycemia and hypertension decreased PCa risk. MetS and obesity did not affect the risk for PCa. However, in multivariate logistic analysis, we found that MetS, hypertension, hyperlipidemia, hyperglycemia, and obesity were not related to the risk of PCa. High age and PSA levels were risk factors for prostate cancer.

**Table 2:** Analysis of risk factors of prostate cancer.

Characteristics	Univariate regression analysis		Multivariate regression analysis	
	OR (95% CI)	P	OR (95% CI)	P
Age	1.110 (1.094, 1.127)	<0.001	1.092 (1.074,1.111)	<0.001
BMI	0.960 (0.898, 1.027)	0.233	–	–
MetS*	1.414 (0.900, 2.221)	0.132	–	–
Obesity*	1.023 (0.691, 1.514)	0.909	–	–
Hypertension*	0.509 (0.344, 0.753)	0.001	1.031 (0.673,1.581)	0.887
Hypertriglyceridaemia*	1.655 (1.089, 2.515)	0.018	0.857 (0.54,1.36)	0.857
Hyperglycaemia*	0.635 (0.428, 0.942)	0.024	0.913 (0.598,1.393)	0.673
Low HDL cholesterol*	2.351 (1.091, 5.067)	0.029	1.559 (0.701,3.466)	0.276
Serum PSA	1.113 (1.095, 1.131)	<0.001	1.078 (1.062,1.094)	<0.001

MetS, metabolic syndrome; PSA, prostate specific antigen; BMI, Body mass index; HDL, high-density lipoprotein. *The definition of MetS is adapted from the modified National Cholesterol Education Programme–Adult Treatment Panel (NCEP-ATP) criteria. Obesity is defined as waist circumference ≥ 85 cm. P < 0.05 was considered significant.

## Discussion

Our study is the first to explore the relationship between MetS and PCa in healthy Chinese people. We found that the incidence of PCa in Chinese men over 40 years of age was 0.1%. We concluded that high age and PSA levels were risk factors for prostate cancer. MetS, hypertension, hyperlipidemia, hyperglycemia, and obesity were not related to the risk of PCa.

MetS is a metabolic disease caused by a series of complex factors, with an incidence of over 34% in the United States (12) and over 21% in China (13). Furthermore, with changes in people’s lifestyles and the adjustment of diets, the global incidence of MetS is increasing (4, 5). A series of studies showed that MetS not only increases the risk for cardiovascular disease (3) but also increases the risk for breast cancer (14), gastric cancer (15), and PCa (16).

The relationship between MetS and PCa has not yet been fully studied, and a review of the published literature does not allow for a definitive conclusion. In a meta-analysis on the impact of MetS on PCa, the results of a regional subgroup analysis showed that MetS was significantly associated with PCa in 8 European studies (RR=1.30, P=0.034), but there was no relationship in 4 U.S. studies (RR=1.03, P=0.390) or 2 Asiatic studies (RR=0.99, P=0.932) (10). A prospective study over a 27-year period in Norway showed that MetS increases the risk for PCa (RR = 1.56, P=0.001) (7). Studies of 2322 Caucasians showed that there is a positive association between MetS and PCa (16). A study of Finnish men revealed that MetS increased the risk for PCa approximately 1.9 times when interference factors such as age, diet and exercise were excluded (P = 0.030) (17). Publications from several European countries have indicated that MetS increases the risk for PCa (7, 16, 17). However, studies from the United States and Australia have shown that MetS can instead reduce the risk for PCa (8, 9). More scholars support the view that although MetS has no significant effect on the incidence of PCa, it increases the risk for high-grade PCa (4, 18).

The reasons for the different conclusions of the studies are mainly as follows. First, different countries have different definition criteria for MetS. For example, Bhindi et al. used the adjusted international joint interim statement in 2009 to define MetS (4). In this study, BMI ≥ 30 instead of abdominal circumference was used to define obesity. However, Tande et al. defined MetS with Adult Treatment Panel III (9). Second, different countries have different levels of economic development, and there are differences in PCa screening levels. For example, countries with lower incomes have a higher incidence of MetS, but they are less likely to screen for PCa (4). Moreover, differences in follow-up times among studies will affect the incidence of PCa. Haheim et al. conducted a prospective study over 27 years and found that MetS increases the risk for PCa (7). Harding et al. found that MetS reduced the incidence of PCa after 8.5 years of follow-up (8). Finally, race affects the risk for PCa with MetS. Beebe-Dimmer et al. found that MetS increased the risk for PCa in African-American men but not in white men (19). Compared with white men, the African-American men who participated in this study had a significantly higher incidence of hypertension and diabetes. This may be a potential biological factor of race affects the MetS, which in turn affects the risk of PCa (19).

The mechanism of the effects of various components of MetS on PCa is still inconclusive. There are several possible explanations for this. MetS leads to abnormal levels of proinflammatory states, insulin-like growth factors, and adipokines (20). Insulin-like growth factor can promote carcinogenic proliferation and delay apoptosis (21). The proinflammatory state causes an increase in cytokines such as tumor necrosis factor, c-reactive protein, interleukin (IL)-6, IL-8, and tumor necrosis factor, which are associated with the risk for PCa (22–24).

We concluded that MetS does not increase the risk for PCa for Chinese. Our results are different from those of other researchers (4, 7–9, 16–18), and there are some explanations for these differences. Our study design was different, and our research was based on a retrospective study of healthy people. For patients with elevated PSA levels or MRI indicating abnormal prostates, we recommended prostate puncture at our hospital and we tracked the Gleason score. Previously published studies were focused on individuals who needed PCa puncture or who had been diagnosed with PCa (4, 7–9, 16–18). In addition, compared with developed countries, Chinese lifestyles are different, and people use different medication interventions (20).

There are some limitations in our present study. First, our study only included Chinese people over the age of 40 who underwent health checkups; thus, our conclusions are not applicable to the overall population. Second, this is a retrospective study, and we may have lost some data, which may have led to bias in our results. Third, some important information, such as smoking, drinking and family history, was not gathered. Finally, the number of health checkups was large, but the numbers of PCa was small. This large gap may have led to the lack of statistically significant associations with some indicators.

## Conclusion

We concluded that high age and PSA levels were risk factors for prostate cancer. MetS, hypertension, hyperlipidemia, hyperglycemia, and obesity were not related to the risk of PCa.

## Data Availability Statement

The original contributions presented in the study are included in the article/supplementary material. Further inquiries can be directed to the corresponding author.

## Ethics Statement

Ethical review and approval was not required for the study on human participants in accordance with the local legislation and institutional requirements. Written informed consent from the participants.

## Author Contributions

XG wrote the manuscript writing. RL collected and analyzed the data. TJ analyzed the data. HT helped design the study and revise article. All authors have read and approved the manuscript.

## Funding

This work was supported by the West China Hospital, Sichuan University/Hospital Management Institute (grant numbers ZX0000075).

## Conflict of Interest

The authors declare that the research was conducted in the absence of any commercial or financial relationships that could be construed as a potential conflict of interest.

## Publisher’s Note

All claims expressed in this article are solely those of the authors and do not necessarily represent those of their affiliated organizations, or those of the publisher, the editors and the reviewers. Any product that may be evaluated in this article, or claim that may be made by its manufacturer, is not guaranteed or endorsed by the publisher.
